# Towards Estimation of HIV-1 Date of Infection: A Time-Continuous IgG-Model Shows That Seroconversion Does Not Occur at the Midpoint between Negative and Positive Tests

**DOI:** 10.1371/journal.pone.0060906

**Published:** 2013-04-16

**Authors:** Helena Skar, Jan Albert, Thomas Leitner

**Affiliations:** 1 Theoretical Biology and Biophysics Group, Los Alamos National Laboratory, Los Alamos, New Mexico, United States of America; 2 Department of Microbiology, Tumor and Cell Biology, Karolinska Institute, Stockholm, Sweden; 3 Clinical Microbiology, Karolinska University Hospital, Stockholm, Sweden; Centro Nacional de Microbiología - Instituto de Salud Carlos III, Spain

## Abstract

Estimating date of infection for HIV-1-infected patients is vital for disease tracking and informed public health decisions, but is difficult to obtain because most patients have an established infection of unknown duration at diagnosis. Previous studies have used HIV-1-specific immunoglobulin G (IgG) levels as measured by the IgG capture BED enzyme immunoassay (BED assay) to indicate if a patient was infected recently, but a time-continuous model has not been available. Therefore, we developed a logistic model of IgG production over time. We used previously published metadata from 792 patients for whom the HIV-1-specific IgG levels had been longitudinally measured using the BED assay. To account for patient variability, we used mixed effects modeling to estimate general population parameters. The typical patient IgG production rate was estimated at *r* = 6.72[approximate 95% CI 6.17,7.33]×10^−3^ OD-n units day^−1^, and the carrying capacity at *K* = 1.84[1.75,1.95] OD-n units, predicting how recently patients seroconverted in the interval ^∧^ *t* = (31,711) days. Final model selection and validation was performed on new BED data from a population of 819 Swedish HIV-1 patients diagnosed in 2002–2010. On an appropriate subset of 350 patients, the best model parameterization had an accuracy of 94% finding a realistic seroconversion date. We found that seroconversion on average is at the midpoint between last negative and first positive HIV-1 test for patients diagnosed in prospective/cohort studies such as those included in the training dataset. In contrast, seroconversion is strongly skewed towards the first positive sample for patients identified by regular public health diagnostic testing as illustrated in the validation dataset. Our model opens the door to more accurate estimates of date of infection for HIV-1 patients, which may facilitate a better understanding of HIV-1 epidemiology on a population level and individualized prevention, such as guidance during contact tracing.

## Introduction

Accurately estimating incidence of an infectious disease is vital for informed and targeted prevention, and knowing the date of infection per case is important for estimating the incidence in a population. For acute infections, like influenza, it is relatively straightforward to infer the date of infection because it occurred just shortly before the diagnosis. For chronic infections, like human immunodeficiency virus type 1 (HIV-1) infection, it is more complicated to infer the date of infection because only rarely are persons diagnosed during primary HIV-1 infection (PHI). Instead, most diagnosed persons have an established HIV-1 infection of unknown duration. Consequently, the World Health Organization (WHO), the Joint United Nations Programme on HIV/AIDS (UNAIDS), as well as national public health institutes usually simply report the number of newly diagnosed cases. Due to the current problems with HIV-1 incidence estimation, there is considerable interest in the development of assays and biomarkers that can determine if an HIV-1 infection is recent, in order to allow for estimating HIV-1 incidence in a population [1], [2], [3], [4], [5], [6], [7], [8].

Seroconversion occurs on average 21 days after HIV-1 infection [9], [10], and is thus a useful date to infer by serology. Serological assays are based on the knowledge about the development and maturation of the HIV-1 antibody response in infected persons (reviewed in [3], [4], [6], [11]). These assays are collectively referred to as Serological Testing Algorithm for Recent HIV Seroconversion (STARHS) [4] or Recent Infection Testing Algorithm (RITA) [2]. In 1998 Janssen et al. described the first mathematical method that was specifically developed to estimate HIV-1 incidence using a cross-sectional sampling approach [12]. This method used results from a “less-sensitive” (or detuned) version and a standard version of an HIV-1 enzyme linked immunoassay (EIA). Since then, additional assays have been developed, such as the IgG capture BED enzyme immunoassay (BED assay) [13], the IDE-V3 assay [14], and several different avidity assays (reviewed in [15]). Adjustments of Janssen’s original formula have also been presented [16], [17]. The BED assay, which was developed by the US Centers for Disease Control and Prevention (CDC), has been commercialized. The assay name ‘BED’ signifies that it is based on a trimeric branched peptide with each branch derived from the immunodominant region of the gp41 glycoprotein of HIV-1 subtype B, circulating recombinant form (CRF) 01_AE or subtype D to overcome subtype-specific differences associated with some other assays [4]. Importantly, the BED assay, like most other serological assays, has been designed for incidence estimates in populations. At present, it provides a binary result, i.e., recent vs. long-term infection based on a cutoff value of a normalized optical density (OD-n = 0.8) in the EIA, rather than a quantitative estimation of time since seroconversion. The mean time interval from seroconversion to this cutoff value, i.e., the mean recency period, has been estimated at around 180 days, with some differences between genetic subtypes and populations [18]. The cutoff value was optimized to minimize misclassification of recent and long-term infections, but such misclassifications still occur. For instance, it is well-established that the BED assay can give a false impression of recent infection for some patients with advanced disease because HIV-1 antibody levels to the BED peptides sometimes wane with advancing immunodeficiency [13]. For this reason different approaches to adjust BED incidence estimates have been suggested [16], [17].

The objectives for this study were: 1) To create a biologically motivated time-continuous model of the production of BED-specific IgG (BED IgG) data; 2) To address the patient variability of the BED IgG growth following HIV-1 seroconversion,; 3) To critically examine the common modeling assumption that sercconversion happens at the midpoint between last negative and first positive HIV-1 test result; and 4) To reevaluate national Swedish surveillance data utilizing BED data. To achieve these goals, we explored various parameterizations of a basic logistic growth model describing the production of BED IgG, trained on a large cohort metadata set from a recent study by Parekh et al. [18]. To account for patient variability, universal parameter values were estimated using mixed effects modeling, and final model selection and validation was performed on a second large dataset, consisting of new BED data from Swedish patients newly diagnosed with HIV-1 infection between 2002 and 2010. While we informed the model with BED assay results, because it is currently the most used biomarker for recency estimation, our model could be adjusted to other available and future serological biomarkers as well as be included in multi-assay approaches [8], [19], [20].

## Materials and Methods

### Ethical Approval

For the new data in this study, collected in Sweden, informed written or oral consent was obtained from all adult participants and from the next of kin, caregivers or guardians on the behalf of participants that were minors or children. The research was conducted according to the Declaration of Helsinki and was approved by the Regional Medical Ethics Board in Stockholm, Sweden, which had permitted the use of oral consent to minimize the risk of selection biases due to patient drop-out because some ethnic groups of participants were known to be willing to take part in the study, but reluctant to provide written consent (Dnr 02–367, 04–797 and 2007/1533). Written or oral consent were documented in the patient clinical records.

### Study Populations and BED Measurements

To infer parameters for our time-continuous IgG model, we used BED data from a previous meta-study by Parekh et al [18], encompassing 756 HIV-1 diagnosed patients sampled at regular intervals in 16 cohorts. These metadata came from longitudinal cohorts where patients were tested on regular intervals with a maximum time span between the last HIV-negative and first HIV-positive test of 365 days (median = 168 days), where the authors assumed that the time of seroconversion occurred at the interval midpoint. We used patients sampled at least twice, resulting in 2975 OD-n measurements from 718 HIV-1 patients.

In addition, we performed BED-testing on plasma samples from 819 patients who were previously diagnosed as HIV-1-infected in Sweden in 2002–2010. These patients are a subset from a recently published study of transmitted drug resistance [21]; we included patients who were living in Sweden when they became infected, whereas patients infected before first arrival in Sweden were not included. The study population constituted 68% (819 of 1196) of all Swedish patients in this category who were diagnosed during the study period and they also accurately reflect the entire population with respect to gender, age, transmission routes, and infections with various HIV-1 subtypes (approximately 40% subtype B, and further subtypes A, C, D, CRF01, CRF02 and others). In contrast to the patients studied by Parekh et al., the Swedish patients did not undergo HIV-testing at regular intervals as they were identified by regular public health diagnostic testing. Nevertheless, for 523 of the 819 Swedish patients we had the date of the last negative HIV-test result.

For the Swedish samples BED OD-n was measured using the Aware™ BED™ EIA HIV-1 Incidence Test (Calypte Biomedical Corporation, Portland, OR, USA) according to the manufacturer’s instructions on a Dynex Technologies MRX Revelation spectrophotometer. A calibrator is used with a known amount of HIV-1-specific IgG in order to make individual runs comparable. Thus, a normalized OD-value (OD-n) for each well is calculated by dividing the raw OD-measurement by the median calibrator value of that individual run. As specified by the manufacturer, samples with OD-n values <1.2 were rerun in triplicate and the median value was used.

### A Logistic Model Describes BED IgG Production

Similar to many biological systems where the rate of reproduction is proportional to the existing population and limited resources, the growth of HIV-1-specific IgG following seroconversion can be modeled by a logistic function,
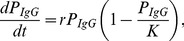
(1)where *r* is the growth rate of HIV-1-specific IgG and *K* is the limiting factor, 
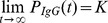
, aka. the carrying capacity. We assume that the carrying capacity does not change over the time we are interested in, i.e., during the ramp-up of IgG directed to HIV-1 in the first few years of infection. The solution to this differential equation is



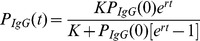



We focus on the portion of HIV-1-specific IgG that is measured by the BED assay [13], [18], [22], henceforth referred to as “BED IgG”. This assay measures the absorbance of light (λ = 450 nm with reference 630–650 nm) of HIV-1-specific IgG complexes. According to Beer-Lambert’s law, the absorbance (optical density, OD) is directly proportional to the concentration of the absorbing species, *OD_IgG_(λ)* = log_10_(*I_0_/I*), where *I_0_/I* is the ratio of light that passes through a solution containing BED IgG complexes. The measured OD*_IgG_* is normalized using an assay standard as described above; the calibrated OD value is denoted OD-n.

Hence, because OD-n operates on a logarithmic scale, the logistic function is transformed to a linear-asymptotic curve, which we model as
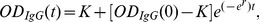
(2)where *r* is modeled as the logarithm to enforce positivity of the rate constant, ensuring it will reach the asymptote *K*.

### Estimating Logistic Model Parameters

**Figure 1:**
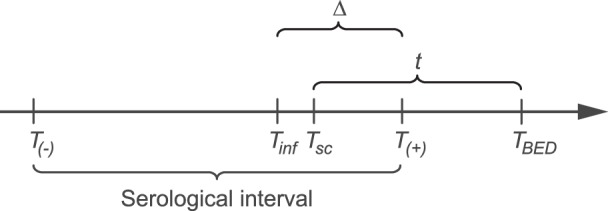
Definitions of dates and time intervals relative to BED testing. The time since seroconversion (*t*) is the time from the date a patient seroconverted (*T*_sc_) to when a sample for BED testing was collected (*T*_BED_). We estimate *t* by a logistic IgG model (Eq. 1) as ^∧^ *t*. Date of infection (*T*_inf_) occurred on average 21 days prior to *T*_sc_ [9], [10]. When available, the patient history also includes the dates of last negative HIV-1 antibody testing (*T*_(−)_) and first positive HIV-1 antibody testing (*T*_(+)_), defining the serological interval. Note that *T_(+)_* and *T_BED_* may often occur at the same date. To reevaluate national Swedish HIV surveillance data we compared ^∧^ *T*_inf_ with *T*_(+)_, resulting in a time difference Δ.

Based on metadata from Parekh et al [18], we selected patients with longitudinal samples (*n*≥2) to use as model training data. In Parekh et al. the mean time interval between their estimated time of seroconversion and reaching a specified assay cutoff value in a population was defined as the “mean recency period”. We are ultimately interested in the date of infection of each patient, *T_inf_(i)*; for that, we first estimate the time (*t)* between when the sample for BED testing was collected (*T*_BED_) and when seroconversion occurred (*T*_sc_), as defined in Figure 1. Thus, the logistic model parameters will refer to time since seroconversion for a typical patient. We used a mixed effects linear-asymptotic model to accommodate the logarithmic OD-n scale to infer *OD_IgG_, r* and *K* (Eq. 2) corresponding to the three parameters of the logistic IgG model (Eq. 1). In addition, we modeled *OD_IgG_* and *r* independently from *K* by a generalized linear mixed effects model in the IgG growth phase (*t*≤350 days).

While we model both the response *OD_IgG_* and the random effects *B* as random variables, we only observe values of *OD_IgG_*. The conditional distribution *OD_IgG_* |*B* and the marginal distribution *B* are independent, multivariate normal distributions. Values of *OD_IgG_* were grouped corresponding to patient, resulting in 2975 OD-n measurements in 718 groups (Figure S1). Consequently, the model estimates parameter values representative for the whole population of the training data in the fixed effects, while the random effects describe conditional modes of the estimated parameters. The random effects in the linear-asymptotic mixed model are described in a general positive-definite matrix structure, allowing parameter inclusion/exclusion as well as defining different covariance dependencies. We tested all possible random effects structures (df = 5–9) to investigate which of them that could be omitted to avoid over-parameterizing our model (Table 1).

**Table 1:** Mixed effects model parameterizations.

Model	*OD(0)*	*r*	*K*	df	AIC
***Full mixed effects model***					
all 3 random effects (ISAR)	1	1	1	9	−
intercept+slope randomeffects (ISR)	1	1	0	7	3648
intercept+asymptote randomeffect (IAR)	1	0	1	7	−
slope+asymptote randomeffects (SAR)	0	1	1	7	3645
intercept random effect (IR)	1	0	0	5	−
slope random effect (SR)	0	1	0	5	3643
asymptote random effect (AR)	0	0	1	5	3971
***Growth phase mixed*** ***effects model***					
correlated growth (CG)	1	1	NA	4	753
uncorrelated growth (UG)	1	1	NA	5	641

Footnotes: 1, random effect included; 0, random effect excluded; −, model did not converge; NA, not applicable.

Similarly, to investigate parameterization level and dependencies in the generalized linear mixed effects modeling of *OD_IgG_* and *r* only, we tested whether a correlated (df = 4) or uncorrelated (df = 5) random effects model would better fit the data. To maximize the amount of patient data and to allow for varying trends as well as varying sampling periods, for this analysis we included all patients sampled at least 5 times within *t* <350 days (N = 116).

All submodels are referred to in the following text by their model abbreviations as defined in Table 1.

The linear-asymptotic mixed model was fitted by full maximum likelihood estimation using the nlme package version 3.1-103 [23] and the generalized linear mixed model was fitted by restricted maximum likelihood (REML) estimation using the lme4 package version 0.999375-42 [24], both made for the R computing environment [25].

### Model Selection, Validation and Estimating Time-bias

To select which of the mixed effects parameterizations best described independent data, model validation was done with the Swedish data. We used our logistic model with parameters estimated by the fixed effects to translate the Swedish OD-n measurements to estimate the time interval (^∧^ *t*) between a patient’s date of seroconversion (^∧^ *T*_sc_) and date of BED test (*T*_BED_). By definition, when OD-n <0.07, the lower BED detection limit [18], ^∧^ *t* = 0. As defined in Figure 1, this time was compared to a time interval constrained by the last negative and first positive HIV-1 testing (the serological interval) relative to BED testing, *T_(−)_* and *T_(+)_,* respectively. Thus, ^∧^ *t* = *α*+*β*|*T_(−)_*+*τ*(*T_(+)_* – *T_(−)_*)|, where τ = (0,1) describes the relative position within the serological interval (*T_(−)_,T_(+)_*), optimized when α = 0 and β = 1, which assumes that ^∧^ *t* perfectly infers *T*_sc_. Final model selection was performed by a hit-and-miss statistic, formally evaluated by a Poisson test. The hit accuracy was measured as the mean distance (in days) between the model-inferred ^∧^ *t*’s and the serological intervals (targets). When a patient’s target was hit the accuracy distance was zero. The precision is then defined as the distribution of the accuracy distances.

### Comparing Date of Infection to National Swedish HIV Surveillance Data

National data on number of diagnoses per year in Sweden were collected from the Swedish Institute for Communicable Disease Control (http://www.smittskyddsinstitutet.se/statistik/hivinfektion/, accessed 01-26-2012). To match the Swedish BED data we excluded patients that had been infected before first arrival to Sweden. Between years 2005–2008 the sampling of our BED data was directly proportional to the national number of diagnoses per year (p>0.05, Wilcoxon rank sum test), and included all relevant transmission risk groups.

The Swedish BED data consisted of patients diagnosed September 2002 through July 2010 (N = 819). The date of infection (*T*_inf_) for each patient was estimated as the date of BED sampling (*T*_BED_) minus two time intervals; the model-inferred time since seroconversion (^∧^ *t*) and a time interval of 21 days between infection and seroconversion. The latter time interval was based on published data on HIV-1 seroconversion phases [9], [10]. Hence, ^∧^ *T*_inf_ = *T*_BED_ – ^∧^ *t* –21 days. To reevaluate national Swedish HIV surveillance data we compared ^∧^ *T*_inf_ with the reported date of diagnosis (first positive HIV-1 sample) *T*_(+)_, resulting in a time difference Δ (Figure 1). As mentioned above, the date of diagnosis and date of BED sampling was identical for many of the patients in our study.

## Results

### Model Training and Parameterizations Using Cohort Metadata

In the full mixed effects modeling the random effect of *OD_IgG_*(0) was found to have very small deviations (model ISR *OD_IgG_*(0) s.d. = 1.22×10^−5^ OD-n units). Furthermore, no covariance between *OD_IgG_*(0) and *r* was observed (model ISR *OD_IgG_*(0) to *r* correlation = 0.001). Similarly, when only modeling *OD_IgG_*(0) and *r* in the IgG growth phase (models CG and UG; Table 1), an ANOVA supported the observation that no pattern of correlation between intercepts *OD_IgG_*(0) and slopes *r* could be observed in the random effects (p<<0.001, χ^2^ = 134.72, df = 1; Table 1). In addition, biologically it is logical to assume that there is no patient variation in the HIV-1-specific IgG level before a patient has been infected; they should all be below detection limit of the BED assay. Indeed, the fixed effect *OD_IgG_*(0) in all models was very close to zero at *t* = 0. Importantly, when *OD_IgG_* = 0, ^∧^ *t* was also close to zero for all models, which means that the assumption that seroconversion on average occurred at the midpoint of the serological interval was correct for these cohort data.

For *K* the relevant time interval since seroconversion is defined by when the BED HIV-1-specific IgG production has reached its asymptote. In the training data that appeared in SAR at approximately ^∧^ *t* = 711 days (OD-n within 99% of *K*) in those patients that were followed at least that long (N = 74). For comparison to the fixed effect *K*, that data resulted in a normally distributed OD-n distribution (p = 0.64, Shapiro-Wilk test), with a weighted mean of OD-n = 2.65 and a standard deviation of 0.89 OD-n units. Thus, our fixed effect *K* is well within the spread of patient data that cover the asymptotic phase, justifying the use of our mixed effects modeling to find the typical patient parameter values.

Overall our best parameterization appeared to be SAR, which includes all fixed effects and the random effects of *r* and *K* (Table 1), however, we could not exclude models SR and ISR on statistical grounds using the cohort training data (AIC scores of SR and ISR were not significantly worse than that of SAR). Model SAR estimated the growth rate at *r* = 0.00672 OD-n units per day, and the asymptote at *K* = 1.85 OD-n units, while models SR and ISR both estimated *r* = 0.00151 and *K* = 3.78. To evaluate which of these models performed best on independent data, we examined new BED data collected from Swedish patients detected by regular public health diagnostic testing, i.e., non-cohort type data.

### Model Selection and Validation Using Non-cohort Type Data

For model validation we used new data from 819 Swedish HIV-1-infected patients diagnosed in 2002–2010. For the SAR model, 500 patients had BED OD-n measurements that fell within the model predictive interval, and of these 350 had a previous negative test. For the SR and ISR models 703 patients had a BED OD-n measurement that fell within the model predictive intervals, and of these 464 had a previous negative test. These patients describe a general population, with different transmission modes, analyzed by one BED measurement per patient, similar to cross-sectional data. A hit-and-miss statistic, measuring whether the model-inferred date of seroconversion(^∧^ *T*_sc_) hit between the dates of collection of the last negative and first positive HIV-1 test (the serological interval; Figure 1), identified SAR as the best model. In the interval where all methods had predictive power (^∧^ *t*<711 days), the point estimate of SAR hit 90% of the patients’ serological intervals, compared to 88% for SR and ISR. When including the 95% confidence interval (CI), SAR hit 94% compared to 92% for SR and ISR. Critically, the number of patient serological intervals SAR predicted correctly while SR and ISR failed was significantly better than when SR and ISR were correct and SAR failed (p<0.05 and p<0.0005, Poisson test, respectively for point estimate and 95% CI estimate). Even when considering the longer predictive interval of SR and ISR (^∧^ *t*<3056 days), these models only hit 86% of the serological intervals.

The accuracy of the estimated date of seroconversion using SAR was on average only 3.8 days off the serological interval, significantly better than SR and ISR at 60 days (p<0.05, paired Wilcoxon rank sum test). Among those patients that SAR missed (N = 29 of 350), unsurprisingly, there was a tendency towards smaller serological intervals (mean target size = 327 days; p<0.001, jackknife subsampling). However, the hit-and-miss statistic for targets <327 days was still good at 88% accuracy. Importantly, SAR showed no correlation between the length of the serological interval (target size) and the precision of the model estimate, measured by the distribution of the accuracy measurements (p = 0.12, Pearson’s correlation = −0.083).

**Figure 2:**
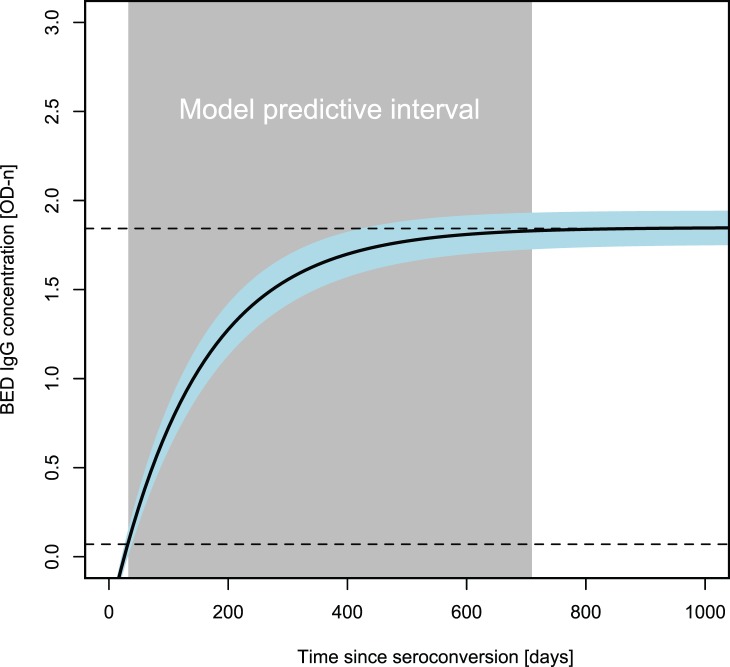
Logistic modeling of IgG-capture BED-enzyme immunoassay absorbance as a function of time since seroconversion. The resulting logistic model is predictive when BED OD-n = (0.07, 1.84), corresponding to 31–711 days. This model describes the typical patient estimated by the SAR mixed effects model (Table 1), where parameter values correspond to the whole population.

Hence, the validation data showed that SAR was our best parameterization of the logistic IgG model (Figure S2), which supports the biological intuition that there is no patient variation in BED OD-n at *t* = 0. Thus, the typical patient is represented by a logistic growth of the BED detected HIV-specific IgG following infection (Figure 2). The model is informative of time since seroconversion when the BED OD-n value of the kit negative control is within an acceptable range of OD-n = (0,0.3), corresponding to ^∧^ *t* = (0,52) days, but specific to each run of BED measurements. Using the average value for a positive test result in the cohort data (OD-n = 0.07 (range: 0.05,0.11; [18]), and OD-n≤1.84 (corresponding to a OD-n value within 99% of the asymptote in SAR), the informative OD-n interval translates into a continuous time interval with predictive power in ^∧^ *t* = (31, 711) days since seroconversion. In our Swedish data the BED kit negative control was at OD-n = 0.16 (range: 0.08,0.28), corresponding to ^∧^ *t* = 39 days. The fixed effects of this model, describing the typical patient with an accuracy of 94%, was described by *OD_IgG_*(0) = −0.35 [−0.40,−0.30] OD-n units, *r* = 6.72 [6.17, 7.33]×10^−3^ OD-n units day^−1^, and the population carrying capacity at *K = *1.84 [1.75, 1.95] OD-n units, where the intervals are the approximate 95% confidence limits (Figure 2).

For comparison, the BED binary classification of whether patients have “recent” or “long-term” HIV-1 infection is based on a cutoff at OD-n = 0.8, reported in different studies to time since seroconversion of 109–220 days [16], [17], [18], [26]. This time overlaps with our time-continuous model (SAR) estimate, which predicts that at OD-n = 0.8 the typical patient seroconverted 92–133 days before BED test sampling (95% CI).

### Date of Seroconversion is Biased Towards Date of Diagnosis

**Figure 3:**
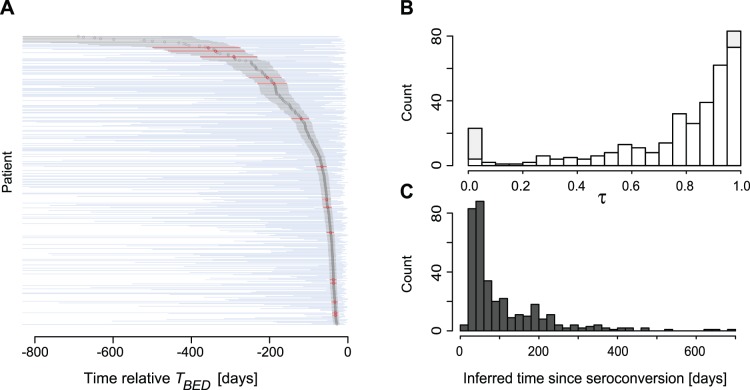
Comparison of inferred time since seroconversion to serological interval. (**A**) The model-inferred time since seroconversion [grey circles with 95% confidence interval as grey lines] from 350 Swedish patients was compared to their known serological intervals [(*T_(+)_,T_(−)_*), blue lines]. When the inferred time since seroconversion did not hit the serological interval, the point estimate and 95% confidence interval is marked in red; 94% of the intervals overlapped. (**B**) The relative positioning parameter τ measures the normalized position of the inferred time since seroconversion to the serological interval. Values outside this interval are shown in grey at τ = 0 and τ = 1. The relative positioning was biased towards the most recent positive HIV test result at large τ. (**C**) Distribution of the inferred time since seroconversion, i.e., the times between BED tests and *τ*-corrected dates of seroconversion.

We next investigated where the inferred date of seroconversion was inferred on each corresponding patient’s serological interval in our Swedish data (N = 350). Naturally, seroconversion (and infection, bar the time from infection to detectable HIV-1 by a valid method [27]) must have happened sometime between the dates that define the serological interval (Figure 1). Recall that the Swedish data was not from a cohort study, but rather data from for patients detected by regular public health diagnostic testing. From this type of data it is not obvious that the population average date of seroconversion is in the middle of the serological interval. Indeed, the Swedish data shows a clear bias of seroconversion shifted towards the date of diagnosis *T*_(+)_(Figure 3). The relative position (τ) of our model-based point-estimate of the date of seroconversion within the serological interval was significantly right-skewed (p<0.01, Wilcoxon rank sum test). Hence, this shows 1) that BED test results are applicable to infer the date of seroconversion in non-cohort type data, but 2) that estimating date of seroconversion as the midpoint between the last negative and the first positive HIV-1 test result is inaccurate and misleading in this type of data.

Patients in the Swedish data (that had a previous negative test and were within the model predictive interval, N = 350) were estimated to have seroconverted at a median of 60 days before BED testing (Figure 3C). However, the long tail of this distribution implies that many patients had seroconverted considerably longer ago. As expected when including patients above the model predictive interval (OD-n>1.84) a longer median time since seroconversion was estimated at 143 days (N = 500). Moreover, when analyzing the entire Swedish set (N = 819), the median time since seroconversion increased to 193 days, with 319 (39%) having an estimated time of seroconversion more than 711 days before sampling. Clearly, to infer date of infection and incidence for entire HIV-infected human populations it becomes important to account for such time intervals.

### Reevaluation of National Swedish HIV Surveillance Data

**Figure 4:**
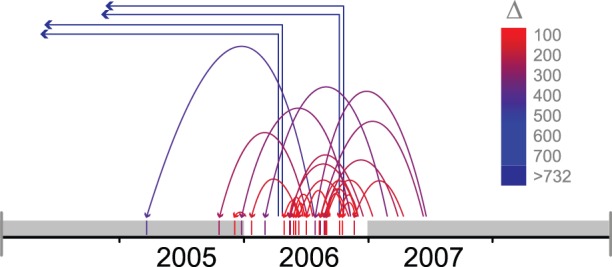
Example from the reevaluation of Swedish IDU surveillance data. Vertical bars of the rug represent inferred date of infection after model application. The maximal time shift, ±732 days, implied by our SAR based predictive upper time-level (711 days) plus average time from infection to seroconversion (21 days) is indicated by grey zones before and after year 2006. The arrows show the resulting shift, starting at the time of diagnosis and pointing at the inferred date of infection, colored according to the time difference Δ.

Using our model-based estimations of date of seroconversion we reevaluated epidemiological data for Swedish patients from whom there previously only was information on the date of diagnosis. As an illustrative example, Figure 4 shows reevaluations affecting year 2006, moving cases into 2006 from following years or out of 2006 to previous years. Most diagnoses stemmed from a date of infection within a year before or after 2006, and a few (n = 4) were estimated to have been infected longer ago than possible to estimate with the SAR model. Note that here the maximal time that can operate is 732 days, composed of the SAR upper predictive value (711 days) plus the average time from infection to seroconversion (21 days).

**Figure 5:**
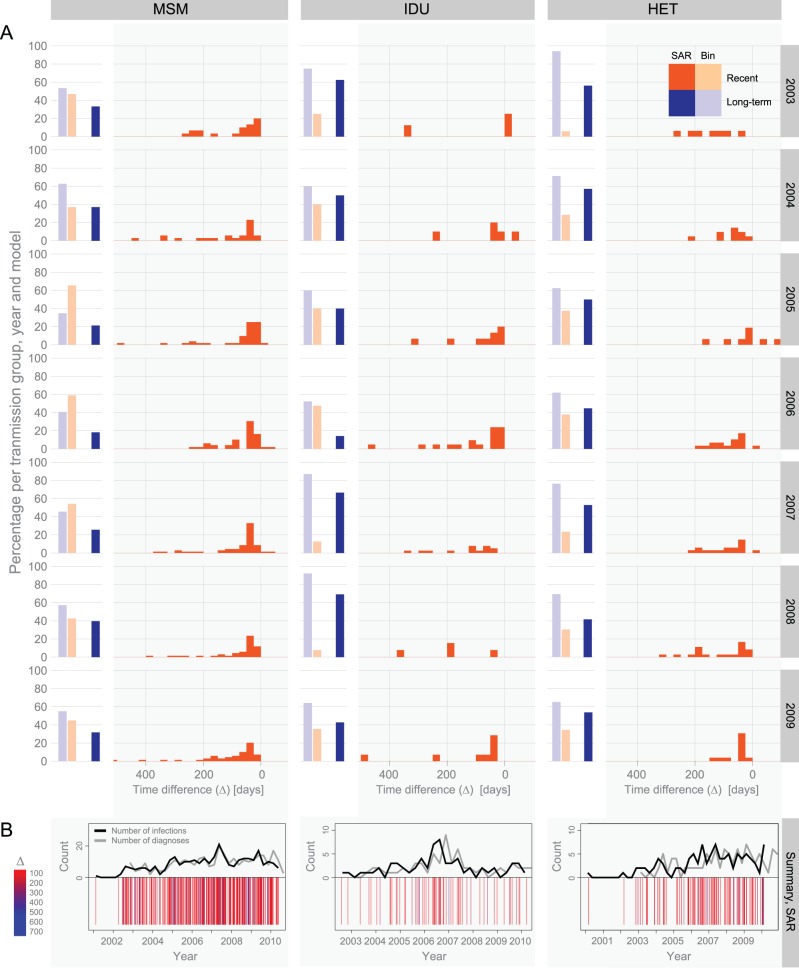
Lattice graph showing date of infection analysis of the Swedish data. (**A**) Time difference (Δ) distributions for the SAR model (dark shades) and Bin (binary) model (light shades) according to transmission group and year. Time differences either fall within (orange) or beyond (blue) the “recent” or quantifiable time since seroconversion, for Bin and SAR respectively. (**B**) Inference of dates of infection according to transmission group. Each bar in the lower part of each graph represents one inferred infection date, colored according to the time difference Δ (same colors as in Figure 4). The upper part of each graph summarizes the number of diagnoses (grey line) and infections (black line) for each quarterly year. For clarity, we have omitted the 95% CI on the bars and curves. See figures 2, 3, and S3 for confidence estimates.

Panel A in Figure 5 shows the resulting distributions of time between diagnosis and date of infection as inferred by our time-continuous IgG model (SAR) for years 2003–2009, partitioned into men who have sex with men (MSM), injecting drug users (IDU), and heterosexual (HET) transmission groups. For comparison, we have included results from conventional BED assay interpretation using a binary model (Bin) which classifies infections as “recent” or “long-term”, with a cutoff at OD-n = 0.8 [18]. Each field shows the predictions of Bin and SAR of patients classified as within (orange) or beyond (blue) the “recent” or quantifiable range, respectively. It is evident that the SAR model gives more informative results, i.e., SAR classifies more patients with its quantifiable range than Bin classifies as “recent”.

Using our date of infection-estimates we note some interesting results relating to the Swedish HIV epidemic: 1) MSM in general had larger proportions of yearly diagnosed individuals that were classified within the quantifiable range (<711 days) than IDU and HET (Figure 5A), suggesting higher risk awareness and more frequent HIV testing within the MSM transmission group in Sweden. 2) In 2006, IDUs showed the greatest deviation in the proportion of yearly diagnoses classified within the quantifiable range (Figure 5A), revealing a change in the epidemic dynamics. 3) Also in IDUs, the most drastic shift resulting from our date of infection-estimates occurred towards 2006 of cases diagnosed in 2007 (Figure 5B), which corresponds to a well-described CRF01_AE outbreak among IDUs in Stockholm discovered in the summer of 2006 [28]. Indeed, further dividing the infections into subtypes showed that the shift was due to CRF01_AE infections and not an increase of subtype B infections, which previously had been the dominantly spreading subtype among Swedish IDUs. Furthermore, the fact that a relatively large number of cases discovered in 2007 were predicted to have been infected for more than 732 days indicates that the outbreak started earlier than the diagnosis dates would suggest.

A comparison of the number of HIV diagnoses (grey line) and SAR inferred date of infections (black line), summarized quarterly from mid-2002 to mid-2010, is shown in Figure 5, Panel B. The lower part of each graph shows the estimated date of infection colored according to the time shift. Only patients that fall within the quantifiable range according to the SAR model are included. The model-inferred infection dates significantly shifted the main IDU outbreak one year back from 2007 to include 2006, as discussed above and shown in Figure S3.

## Discussion

Due to the current limitations in HIV-1 incidence estimation there is considerable interest amongst international and national public health agencies in serological biomarkers that relate to the time of HIV-1 infection, such as the BED assay that was developed by the USA CDC [1], [2], [3], [4], [5], [6], [12], [13], [29], [30], [31]. For example, an international panel recently urged for novel incidence assays and algorithms, especially for use on cross-sectional data [32]. Here, we have created a time-continuous model that allows quantitative estimation of time since seroconversion based on BED assay results that works on both cohort and cross-sectional type of data. We expect that time-continuous rather than two-level discrete (recent or long-term) estimates will make incidence estimation more accurate, given that this new detailed information can be incorporated in algorithms used to calculate incidence in a population. For instance, Sommen et al [33] recently proposed an approach for estimating HIV incidence from continuous biomarker values. We exemplify that our quantitative estimation of time since seroconversion can improve national Swedish HIV surveillance data, which currently show a peak in newly diagnosed cases in 2007 while our analyses show that most of the corresponding infections occurred in 2006. Importantly, this finding is corroborated by independent phylodynamic analyses of an outbreak among IDUs [28]. Our method should be valuable also in other countries that have incorporated biomarker testing in their national HIV surveillance programs, e.g., France, the UK and Germany [6], [30], [31].

For patients diagnosed as a result of seeking public health diagnostic testing services we found that the date of seroconversion, as inferred by our BED-based SAR model, was closer to the first HIV-1 positive sample than to the midpoint between the last negative and first positive sample. This should not be surprising because persons who seek public health services often have a reason to get tested for HIV infection, such as an unsafe sexual encounter. In contrast, we found that midpoint dating is a valid approach for cohort population-studies of HIV-1 infection (followed longitudinally) because they are tested at regular intervals rather than due to perceived risk of infection. Hence, date of serocoversion, and by inference date of infection, can be estimated using the midpoint approach for cohort data, but not for data resulting from public health diagnostic testing. This fact is supported by previous incidence modeling results showing that cohort-based estimates were robust against dependence between testing and time of infection, while STARHS estimates may be biased because of early testing in recently infected persons [34], [35]. This finding is relevant because the “midpoint assumption” frequently is made also on data collected from patients diagnosed in public health services; this includes for instance the three large European collaborative projects Eurosida, Cascade, and Spread [36], [37], [38], [39].

Every natural system is limited by its resources. The HIV-specific IgG growth within a patient thus has to reach a limit, in population biology often referred to as the carrying capacity of the system. Our model does not specify the limiting factor(s), but it is obviously related to the immune response to HIV replication and production, which in turn is controlled by e.g. target cell populations and immune clearance [40]. Thus, this becomes a dynamic system that ultimately determines the carrying capacity of the system. Over long infection times, and certainly with development of immunodeficiency, it is reasonable to assume that the carrying capacity changes. While logistic models with more than one carrying capacity have been developed [41], [42], we did not include this complication as we are only interested in the stage during which the initial IgG response develops. Once the (first) carrying capacity is reached (here at *t* >711 days), such models no longer have power to predict time. Encouragingly, based on more limited data, another paper published while our study was under review found a similar expression for the BED IgG growth using a statistical approach rather than our biologically principled approach [43]. Similar to the biological limiting factors that influence the carrying capacity, the OD measurements also have an upper limit. Most ELISA spectrophotometers used to measure absorbance have an upper limit of OD = 3–4, and therefore there is also a technical limit on the maximum IgG concentration that can be measured. Note that this refers to the raw OD measured directly on each sample. In the BED assay, the raw measurements are normalized by an assay standard, making results comparable between runs. This standard should have a raw absorbance in the interval OD = (0.380,1.350), resulting in an upper OD-n range that can be reliably modeled of 2.22–10.53. As our model estimates the carrying capacity at *K = *1.84 [1.75, 1.95] OD-n units, instrument limitations should have minimal impact in the model predictive interval of ^∧^ *t*≤711 days. For patients with high OD-n values, it is possible that the model predictive interval could be extended by serial dilutions of patient’s serum samples prior to BED testing, but this is something that we have not yet explored, partly because no such training data are available.

Recently, Parekh et al showed that different human populations as well as humans infected with different HIV-1 subtypes may show different rates of development of BED-specific IgG in response to HIV-1 infection [18]. They analyzed a large set of cohorts from different geographical locations worldwide, and concluded that previous recency period cutoff-times based on subtype B virus infections needed to be adjusted to better describe world variation. The new BED kit instructions will be updated to reflect this important finding. The parameter values of our logistic model were informed by a slightly expanded set, kindly provided by Dr. Parekh, and thus also reflect the world human- as well as HIV-1-variation. Similarly, our Swedish data also consisted of patients of different genetic backgrounds as well as infections with different HIV-1 subtypes [21], [44]. However, we did not attempt to explicitly include data about human genetics or HIV-1 genetic subtype in our model because 1) it is still largely unknown if human genetic factors involved in humoral immune responses differ among human populations, 2) differences within HIV-1 subtypes appear to affect OD-n trends as much as between subtypes [18], and 3) often this type of information is not available anyway.

The fact that the BED assay can give a false impression of recent infection for patients with advanced disease [13] deserves some discussion. This is an important problem when the BED assay is used for HIV-incidence estimation in populations by anonymous testing. Thus, if diagnosis occurs in late stage infection and no other clinical data is available, e.g. CD4 counts, the problem with false recent classification becomes more severe. However, for our Swedish patients we had access to CD4 counts informing about possible late stage. We are currently exploring if our model can be further improved by formal incorporation of CD4 counts as a covariate and/or by using results from two or more consecutive BED-tests from each patient. Similarly, “late presentation”, i.e. persons presenting for care with a CD4 count below 350 cells/mL [45], [46], affects around 50% of patients diagnosed in several European and US settings [45], [47], [48], [49]. Late presentation is an important clinical problem because it leads to increased morbidity and mortality [45], [46], [50] as well as epidemiological problems because patients who are unaware of their infection are more likely to transmit the infection to others than patients who have been diagnosed [51]. However, it is important to point out that late presentation is not equivalent to a long-standing infection [52]. Thus, our method to estimate the date of infection could add important information on the epidemiology of late presentation.

In conclusion, we have created a model that quantifies the time since seroconversion based on a simple serological assay, i.e. the BED assay. The model is applicable to BED results from patients included in cohort studies as well as patients diagnosed as a result of public health services. This model should be generally applicable to many quantitative antibody tests, such as improved HIV-1 “recency”-tests as well as tests for other pathogens. We show that using the midpoint between the last negative and the first positive HIV-1 sample gives an inaccurate estimate of date of seroconversion for patients identified by regular public health diagnostic testing, but has validity for patients who are sampled at pre-defined intervals, e.g. in longitudinal cohort studies. We expect that our method can improve incidence estimates, and thus provide valuable information for HIV-1 surveillance and prevention.

## Supporting Information

Figure S1 **Model training data.** The graph shows data from Parekh et al [18] for patients sampled ≥2 times. This formed the model training data and included 2975 OD-n measurements from 718 patients.
(PDF)

Figure S2 **Individual patient data compared to population estimate.** Each of the 718 patient’s BED data in the model training set is individually compared to our logistic IgG model (Eq. 1) informed by the SAR-estimated fixed effects parameter values (blue lines).
(PDF)

Figure S3 **The difference of estimating date of infection compared to using date of diagnosis.** The 95% confidence bands of the difference between number of diagnoses and estimated infections shown quarterly for the IDU transmission group. Points on the zero line indicate that the number of diagnoses is a good approximation of the number of infections in that quarter. A significant deviation from zero is highlighted with red points when diagnoses would underestimate infections and blue points for overestimating infections.
(EPS)

## References

[1] Incidence Assay Critical Path Working Group (2011) More and better information to tackle HIV epidemics: towards improved HIV incidence assays. PLoS medicine 8: e1001045. 21731474 10.1371/journal.pmed.1001045 PMC3114871

[2] UNAIDS/WHO Working Group on Global HIV/AIDS and STI Surveillance (2011) When and how to use assays for recent infection to estimate HIV incidence at a population level. Geneva, Switzerland: World Health Organization.

[3] Busch MP, Pilcher CD, Mastro TD, Kaldor J, Vercauteren G, et al (2010) Beyond detuning: 10 years of progress and new challenges in the development and application of assays for HIV incidence estimation. AIDS 24: 2763–2771. 20975514 10.1097/QAD.0b013e32833f1142

[4] Murphy G, Parry JV (2008) Assays for the detection of recent infections with human immunodeficiency virus type 1. Euro Surveill 13. 18775293

[5] Park SY, Love TM, Nelson J, Thurston SW, Perelson AS, et al (2011) Designing a genome-based HIV incidence assay with high sensitivity and specificity. AIDS 25: F13–19. 21716075 10.1097/QAD.0b013e328349f089 PMC3319025

[6] Le Vu S, Pillonel J, Semaille C, Bernillon P, Le Strat Y, et al.. (2008) Principles and uses of HIV incidence estimation from recent infection testing–a review. Euro surveillance : bulletin europeen sur les maladies transmissibles = European communicable disease bulletin 13. 18775292

[7] Hallett TB (2011) Estimating the HIV incidence rate: recent and future developments. Current opinion in HIV and AIDS 6: 102–107. 21505383 10.1097/COH.0b013e328343bfdb PMC3083833

[8] Laeyendecker O, Brookmeyer R, Cousins MM, Mullis CE, Konikoff J, et al.. (2012) HIV Incidence Determination in the United States: A Multi-Assay Approach. The Journal of infectious diseases. 10.1093/infdis/jis659 PMC3532826 23129760

[9] Fiebig EW, Wright DJ, Rawal BD, Garrett PE, Schumacher RT, et al (2003) Dynamics of HIV viremia and antibody seroconversion in plasma donors: implications for diagnosis and staging of primary HIV infection. AIDS 17: 1871–1879. 12960819 10.1097/00002030-200309050-00005

[10] Cohen MS, Shaw GM, McMichael AJ, Haynes BF (2011) Acute HIV-1 Infection. The New England Journal of Medicine 364: 1943–1954. 21591946 10.1056/NEJMra1011874 PMC3771113

[11] Guy R, Gold J, Calleja JM, Kim AA, Parekh B, et al (2009) Accuracy of serological assays for detection of recent infection with HIV and estimation of population incidence: a systematic review. Lancet Infect Dis 9: 747–759. 19926035 10.1016/S1473-3099(09)70300-7

[12] Janssen RS, Satten GA, Stramer SL, Rawal BD, O’Brien TR, et al (1998) New testing strategy to detect early HIV-1 infection for use in incidence estimates and for clinical and prevention purposes. JAMA 280: 42–48. 9660362 10.1001/jama.280.1.42

[13] Dobbs T, Kennedy S, Pau CP, McDougal JS, Parekh BS (2004) Performance characteristics of the immunoglobulin G-capture BED-enzyme immunoassay, an assay to detect recent human immunodeficiency virus type 1 seroconversion. Journal of Clinical Microbiology 42: 2623–2628. 15184443 10.1128/JCM.42.6.2623-2628.2004 PMC427871

[14] Barin F, Meyer L, Lancar R, Deveau C, Gharib M, et al (2005) Development and validation of an immunoassay for identification of recent human immunodeficiency virus type 1 infections and its use on dried serum spots. J Clin Microbiol 43: 4441–4447. 16145089 10.1128/JCM.43.9.4441-4447.2005 PMC1234099

[15] Murphy G, Parry JV (2008) Assays for the detection of recent infections with human immunodeficiency virus type 1. Euro surveillance : bulletin europeen sur les maladies transmissibles = European communicable disease bulletin 13. 18775293

[16] Hargrove JW, Humphrey JH, Mutasa K, Parekh BS, McDougal JS, et al (2008) Improved HIV-1 incidence estimates using the BED capture enzyme immunoassay. AIDS 22: 511–518. 18301064 10.1097/QAD.0b013e3282f2a960

[17] McDougal JS, Parekh BS, Peterson ML, Branson BM, Dobbs T, et al (2006) Comparison of HIV type 1 incidence observed during longitudinal follow-up with incidence estimated by cross-sectional analysis using the BED capture enzyme immunoassay. AIDS Res Hum Retroviruses 22: 945–952. 17067263 10.1089/aid.2006.22.945

[18] Parekh BS, Hanson DL, Hargrove J, Branson B, Green T, et al (2011) Determination of mean recency period for estimation of HIV type 1 Incidence with the BED-capture EIA in persons infected with diverse subtypes. AIDS Research and Human Retroviruses 27: 265–273. 20954834 10.1089/aid.2010.0159

[19] Duong YT, Qiu M, De AK, Jackson K, Dobbs T, et al (2012) Detection of recent HIV-1 infection using a new limiting-antigen avidity assay: potential for HIV-1 incidence estimates and avidity maturation studies. PLoS ONE 7: e33328. 22479384 10.1371/journal.pone.0033328 PMC3314002

[20] Ragonnet-Cronin M, Aris-Brosou S, Joanisse I, Merks H, Vallee D, et al (2012) Genetic diversity as a marker for timing infection in HIV-infected patients: evaluation of a 6-month window and comparison with BED. The Journal of infectious diseases 206: 756–764. 22826337 10.1093/infdis/jis411

[21] Karlsson A, Bjorkman P, Bratt G, Ekvall H, Gisslen M, et al.. (2012) Low prevalence of transmitted drug resistance in patients newly diagnosed with HIV-1 infection in Sweden 2003–2010. PLoS ONE: in press. 10.1371/journal.pone.0033484 PMC3308981 22448246

[22] Parekh BS, Kennedy MS, Dobbs T, Pau CP, Byers R, et al (2002) Quantitative detection of increasing HIV type 1 antibodies after seroconversion: a simple assay for detecting recent HIV infection and estimating incidence. AIDS Research and Human Retroviruses 18: 295–307. 11860677 10.1089/088922202753472874

[23] Pinheiro J, Bates D, DebRoy S, Sarkar D, the R Core team (2012) nlme: Linear and nonlinear mixed effects models. 3.1–103 ed.

[24] Bates D, Maechler M, Bolker B (2011) lme4: Linear mixed-effects models using S4 classes. 0.999375–42 ed.

[25] R Development Core Team (2003) R: A language and environment for statistical computing. Vienna, Austria: R Foundation for Statistical Computing.

[26] Karita E, Price M, Hunter E, Chomba E, Allen S, et al (2007) Investigating the utility of the HIV-1 BED capture enzyme immunoassay using cross-sectional and longitudinal seroconverter specimens from Africa. AIDS 21: 403–408. 17301558 10.1097/QAD.0b013e32801481b7

[27] Song R, Karon JM, White E, Goldbaum G (2006) Estimating the distribution of a renewal process from times at which events from an independent process are detected. Biometrics 62: 838–846. 16984327 10.1111/j.1541-0420.2006.00536.x

[28] Skar H, Axelsson M, Berggren I, Thalme A, Gyllensten K, et al (2011) Dynamics of two separate but linked HIV-1 CRF01_AE outbreaks among injection drug users in Stockholm, Sweden, and Helsinki, Finland. Journal of Virology 85: 510–518. 20962100 10.1128/JVI.01413-10 PMC3014206

[29] Von Gegerfelt A, Albert J, Morfeldt-Manson L, Broliden K, Fenyo EM (1991) Isolate-specific neutralizing antibodies in patients with progressive HIV-1-related disease. Virology 185: 162–168. 1926772 10.1016/0042-6822(91)90764-3

[30] Fisher M, Pao D, Murphy G, Dean G, McElborough D, et al (2007) Serological testing algorithm shows rising HIV incidence in a UK cohort of men who have sex with men: 10 years application. AIDS 21: 2309–2314. 18090279 10.1097/QAD.0b013e3282ef9fed

[31] Batzing-Feigenbaum J, Kollan C, Kuhne A, Matysiak-Klose D, Gunsenheimer-Bartmeyer B, et al (2011) Cohort profile: the German ClinSurv HIV project–a multicentre open clinical cohort study supplementing national HIV surveillance. HIV medicine 12: 269–278. 20955355 10.1111/j.1468-1293.2010.00879.x

[32] Sharma U, Schito M, Welte A, Rousseau C, Fitzgibbon J, et al.. (2011) Novel Biomarkers for HIV Incidence Assay Development. AIDS Research and Human Retroviruses. 10.1089/aid.2011.0332 PMC3358102 22206265

[33] Sommen C, Commenges D, Vu SL, Meyer L, Alioum A (2011) Estimation of the distribution of infection times using longitudinal serological markers of HIV: implications for the estimation of HIV incidence. Biometrics 67: 467–475. 20731647 10.1111/j.1541-0420.2010.01473.x

[34] White EW, Lumley T, Goodreau SM, Goldbaum G, Hawes SE (2010) Stochastic models to demonstrate the effect of motivated testing on HIV incidence estimates using the serological testing algorithm for recent HIV seroconversion (STARHS). Sexually transmitted infections 86: 506–511. 21062766 10.1136/sti.2009.037481 PMC3425390

[35] Remis RS, Palmer RW (2009) Testing bias in calculating HIV incidence from the Serologic Testing Algorithm for Recent HIV Seroconversion. AIDS 23: 493–503. 19240458 10.1097/QAD.0b013e328323ad5f

[36] Vercauteren J, Wensing AM, van de Vijver DA, Albert J, Balotta C, et al (2009) Transmission of drug-resistant HIV-1 is stabilizing in Europe. The Journal of infectious diseases 200: 1503–1508. 19835478 10.1086/644505

[37] Collaborative Group on AIDS Incubation and HIV Survival including the CASCADE EU Concerted Action (2000) Time from HIV-1 seroconversion to AIDS and death before widespread use of highly-active antiretroviral therapy: a collaborative re-analysis. Collaborative Group on AIDS Incubation and HIV Survival including the CASCADE EU Concerted Action. Concerted Action on SeroConversion to AIDS and Death in Europe. Lancet 355: 1131–1137. 10791375

[38] Bannister WP, Cozzi-Lepri A, Clotet B, Mocroft A, Kjaer J, et al (2008) Transmitted drug resistant HIV-1 and association with virologic and CD4 cell count response to combination antiretroviral therapy in the EuroSIDA Study. Journal of acquired immune deficiency syndromes 48: 324–333. 18545152 10.1097/QAI.0b013e31817ae5c0

[39] Cascade collaboration (2003) Determinants of survival following HIV-1 seroconversion after the introduction of HAART. Lancet 362: 1267–1274. 14575971 10.1016/s0140-6736(03)14570-9

[40] Perelson AS, Neuman AU, Markowitz M, Leonard JM, Ho DD (1996) HIV-1 dynamics in vivo: virion clearance rate, infected cell life-span, and viral generation time. Science 271: 1582–1586. 8599114 10.1126/science.271.5255.1582

[41] Roughgarden J (1974) Population dynamics in a spatially varying environment: How population size “tracks” spatial variation in carrying capacity. American Naturalist 108: 649–664.

[42] Meyer PS, Ausubel JH (1999) Carrying capacity: a model with logistically varying limits. Technological Forecasting and Social Change 61: 209–214.

[43] Hargrove J, Eastwood H, Mahiane G, van Schalkwyk C (2012) How should we best estimate the mean recency duration for the BED method? PLoS ONE 7: e49661. 23166743 10.1371/journal.pone.0049661 PMC3500313

[44] Alaeus A, Leitner T, Lidman K, Albert J (1997) Most genetic subtypes of HIV-1 have entered Sweden. AIDS 11: 199–202. 9030367 10.1097/00002030-199702000-00010

[45] Sabin CA, Schwenk A, Johnson MA, Gazzard B, Fisher M, et al (2010) Late diagnosis in the HAART era: proposed common definitions and associations with mortality. AIDS 24: 723–727. 20057312 10.1097/QAD.0b013e328333fa0f

[46] Antinori A, Coenen T, Costagiola D, Dedes N, Ellefson M, et al (2011) Late presentation of HIV infection: a consensus definition. HIV medicine 12: 61–64. 20561080 10.1111/j.1468-1293.2010.00857.x

[47] Zoufaly A, an der Heiden M, Marcus U, Hoffmann C, Stellbrink H, et al (2012) Late presentation for HIV diagnosis and care in Germany. HIV medicine 13: 172–181. 22093171 10.1111/j.1468-1293.2011.00958.x

[48] d’Arminio Monforte A, Cozzi-Lepri A, Girardi E, Castagna A, Mussini C, et al (2011) Late presenters in new HIV diagnoses from an Italian cohort of HIV-infected patients: prevalence and clinical outcome. Antiviral therapy 16: 1103–1112. 22024526 10.3851/IMP1883

[49] Centers for Disease Control and Prevention (CDC) (2009) Late HIV testing - 34 states, 1996–2005. MMWR Morbidity and mortality weekly report 58: 661–665. 19553901

[50] Kitahata MM, Gange SJ, Abraham AG, Merriman B, Saag MS, et al (2009) Effect of early versus deferred antiretroviral therapy for HIV on survival. The New England Journal of Medicine 360: 1815–1826. 19339714 10.1056/NEJMoa0807252 PMC2854555

[51] Marks G, Crepaz N, Senterfitt JW, Janssen RS (2005) Meta-analysis of high-risk sexual behavior in persons aware and unaware they are infected with HIV in the United States: implications for HIV prevention programs. Journal of acquired immune deficiency syndromes 39: 446–453. 16010168 10.1097/01.qai.0000151079.33935.79

[52] Lodi S, Phillips A, Touloumi G, Geskus R, Meyer L, et al (2011) Time from human immunodeficiency virus seroconversion to reaching CD4+ cell count thresholds <200, <350, and <500 Cells/mm(3): assessment of need following changes in treatment guidelines. Clinical infectious diseases : an official publication of the Infectious Diseases Society of America 53: 817–825. 21921225 10.1093/cid/cir494

