# Supplementary figures and images for: Towards Estimation of HIV-1 Date of Infection: A Time-Continuous IgG-Model Shows That Seroconversion Does Not Occur at the Midpoint between Negative and Positive Tests
Source: PLoS One. 2013 Apr 16;8(4):e60906. doi: 10.1371/journal.pone.0060906 (PMC3628711; doi:10.1371/journal.pone.0060906)

Cohort metadata [  $\geq 2$  datapoints/patient; No. patients = 718 ]

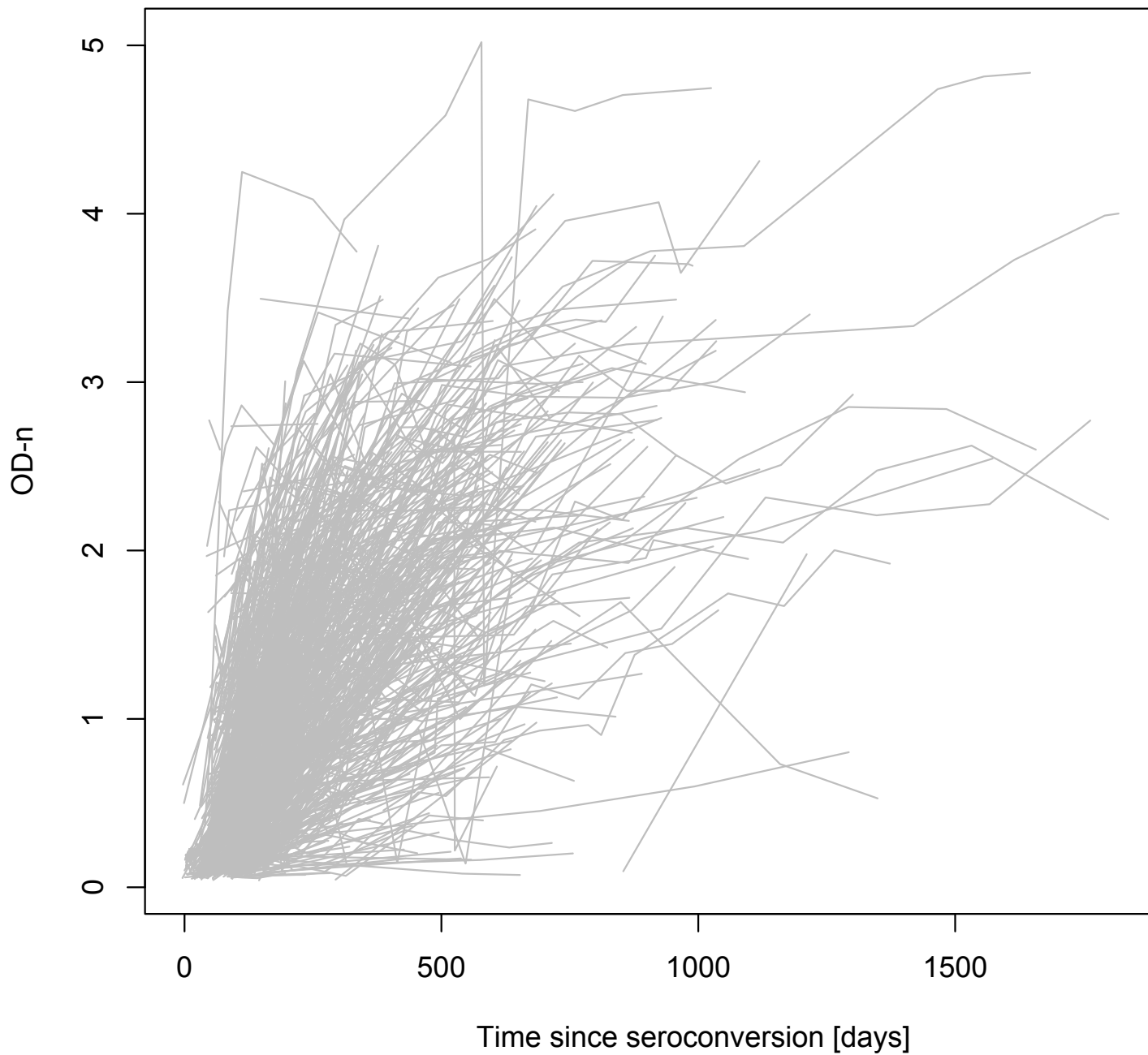

Supplement: Figure S1 — Model training data. The graph shows data from Parekh et al [18] for patients sampled ≥2 times. This formed the model training data and included 2975 OD-n measurements from 718 patients. (PDF) [file pone.0060906.s001.pdf]

OD-n

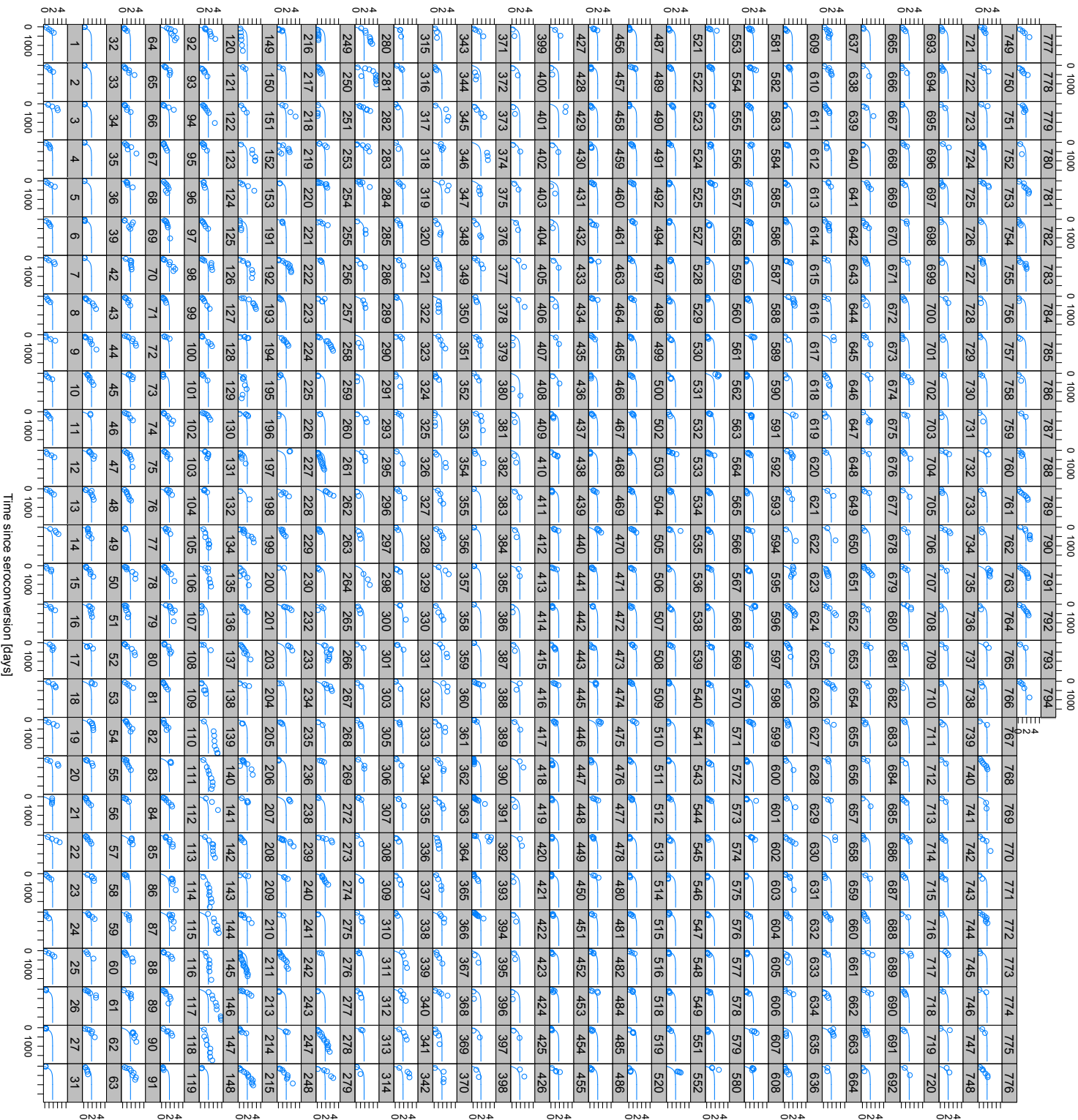

Supplement: Figure S2 — Individual patient data compared to population estimate. Each of the 718 patient’s BED data in the model training set is individually compared to our logistic IgG model (Eq. 1) informed by the SAR-estimated fixed effects parameter values (blue lines). (PDF) [file pone.0060906.s002.pdf]
